# Using American Sign Language–Fluent Community Health Navigators to Advance Cancer Screening Adherence through Videoconferencing With Deaf, Deafblind, and Hard of Hearing Adults: Protocol for a Randomized Controlled Trial

**DOI:** 10.2196/65078

**Published:** 2025-09-09

**Authors:** Poorna Kushalnagar, Sowmya R Rao, Erika Bergeron, Rupa Valdez, Regina Wang, Raja S Kushalnagar, Georgia R Sadler

**Affiliations:** 1 Center of Deaf Health Excellence Gallaudet University Washington, DC United States; 2 School of Public Health Boston University Boston, MA United States; 3 Department of Public Health Sciences University of Virginia Charlottesville, VA United States; 4 UC San Diego Health System La Jolla, CA United States; 5 School of Science, Technology, Accessibility, Mathematics, and Public Health Gallaudet University Washington, DC United States; 6 Moores Cancer Center University of California, San Diego La Jolla, CA United States

**Keywords:** deaf, sign language, cancer screening, adherence, hearing loss

## Abstract

**Background:**

Cancer screening nonadherence persists among adults who are deaf, deafblind, and hard of hearing (DDBHH). These barriers span individual, clinician, and health care system levels, contributing to difficulties understanding cancer information, accessing screening services, and following treatment directives. Critical communication barriers include ineffective patient-physician communication, limited access to American Sign Language (ASL) cancer information, misconceptions about medical procedures, insurance navigation difficulties, and intersectional barriers for multiply marginalized individuals.

**Objective:**

This randomized controlled trial addresses these barriers by implementing the first videoconference-based study of ASL-fluent community health navigators (ASL-CHNs) to improve cancer screening adherence among adults who are DDBHH. The study tests whether ASL-CHN intervention results in greater adherence to cancer screening guidelines, improved patient-physician communication ratings, and increased cancer knowledge compared to standard care.

**Methods:**

The study uses a videoconference-delivered, block-randomized design stratifying 200 participants who are DDBHH by age and sex, with 100 participants assigned to the ASL-CHN intervention and 100 to standard care. All participants are confirmed as nonadherent to at least 1 of 5 age-appropriate cancer screening guidelines recommended by the United States Preventive Services Task Force for breast, cervical, colorectal, lung, and prostate cancers. Recruitment occurred nationwide through multiple strategies including prior study participants, community partners, and major community events. The intervention arm receives support from specially trained ASL-CHNs over several months, accommodating lengthy scheduling processes for cancer screenings. Primary outcomes measure completion of age- and risk-appropriate cancer screening, with prostate cancer focusing on shared decision-making participation. Secondary outcomes assess patient-physician communication using the validated National Cancer Institute’s Health Information National Trends Survey (NCI-HINTS) Patient Centered Communication questionnaire in ASL. Tertiary outcomes examine cancer knowledge through validated measures. The analysis uses intent-to-treat methodology using multivariable logistic regression, accounting for potential clustering effects and anticipated 25% attrition.

**Results:**

As of August 2025, more than 75% of the target enrollment has been achieved. Preliminary data indicate that the intervention group is consistently outperforming the standard care group in cancer screening adherence, supporting the study hypothesis that ASL-CHNs are effective in promoting cancer screening adherence among previously nonadherent participants who are DDBHH.

**Conclusions:**

The ASL-CHN intervention represents an accessible, scalable solution for reducing cancer screening disparities. By combining personalized navigation with ASL-fluent community health support through videoconferencing, this intervention addresses limitations of previous screening programs that lacked accessible support. If successful, the ASL-CHN model could provide health care providers with a practical, recommendable option for patients who are DDBHH requiring navigator support that can be done remotely through videoconferencing, potentially improving early detection rates and reducing cancer mortality in this underserved population while advancing accessible care delivery.

**Trial Registration:**

ClinicalTrials.gov NCT06492993; https://clinicaltrials.gov/study/NCT06492993

**International Registered Report Identifier (IRRID):**

DERR1-10.2196/65078

## Introduction

### Background

Cancer health disparities are common issues across underserved populations that are driven by social determinants of health. As detailed in *Community Health Navigators for Cancer Screening among Deaf, Deafblind, and Hard of Hearing (DDBHH) Adults Who Use American Sign Language* [1], the barriers to health screening and treatment are multilevel, persisting at the individual, clinician, and health care system levels, and can affect patients who are deaf, deafblind, and hard of hearing (DDBHH) across their lifespan. There are documented reports of difficulties in understanding cancer health information in print [1-3], accessing clinical services for screenings [1,4-7], and understanding and following treatment-related directives if the screening results in a cancer diagnosis [8]. Communication difficulties contribute to delayed diagnoses, exclusion from clinical trials, and differential treatment that result in poorer cancer outcomes for deaf and hard of hearing patients.[9]. The complexity of the field of oncology and rapidly expanding associated vocabulary further exacerbates these health inequities. Despite the availability of basic cancer information in American Sign Language (ASL) that helps improve cancer literacy for people who use ASL, the complex nature of cancer prevention, detection, and treatment creates barriers to care for individuals who are DDBHH that even exceed the challenges of hearing non-English speaking populations. The current standard of care for individuals who are DDBHH may include visiting doctors alone, with a sign language interpreter who may or may not be capable of interpreting at the level needed in the complex oncology environment, or with a family member.

Community health workers (CHW) and navigators (CHN) are becoming more common in health care systems, based on research that confirms their ability to help patients more successfully navigate through their health care system [10]. For example, the Baltimore HEARS project demonstrated the success of a CHW-delivered intervention program for older adults who already had considerable spoken and written language proficiency because their hearing loss occurred later in life [10]. The CHW hearing loss program relied upon materials that are not easily accessible to people who rely upon ASL as their preferred form of communication. Thus, the focus of this research project was to create and evaluate a training program to prepare CHNs to work with individuals who are DDBHH, the majority of whom use ASL as their primary means of communication and have limited English language skills.

For the vulnerable group of people who are DDBHH in need of cancer screening and preventive care, an intervention delivered by CHNs who use ASL and ASL-based teaching materials offers an excellent source of relief and support in the face of barriers to health care. This paper describes the development and evaluation of a CHN intervention that is accessible in ASL as a logical strategy for reducing barriers to improved health care access for people who are DDBHH.

### Trial Aim

The aim of this trial is to implement the first randomized controlled trial of a DDBHH CHN intervention that is (1) accessible in ASL and English, (2) easy to understand, (3) relevant to the experiences of people who are DDBHH, (4) inclusive of videoconferencing for face-to-face ASL communication, and (5) easy to use. The intervention will be evaluated using multiple criteria to assess the effectiveness of the navigator in guiding patients who are DDBHH through the cancer screening process.

### Hypothesis

This study tested the hypothesis that the use of ASL-fluent CHNs (ASL-CHNs) will result in:

1. Patients who are DDBHH demonstrating greater adherence to cancer screening guidelines compared to those assigned to the standard of care group (without access to this program’s trained CHNs).
2. Higher patient-physician communication ratings by patients who are DDBHH compared to those assigned to the standard of care group.
3. A direct relationship between increased cancer knowledge and cancer screening adherence compared to the standard of care group.

## Methods

### Ethical Considerations

The protocol (IRB-FY21-120) was reviewed and approved by Gallaudet University’s Institutional Review Board (IRB) prior to recruitment.

Eligible participants undergo a consenting process with an ASL-fluent clinical research coordinator who explains the study purpose and expectations in both English (fourth grade reading level) and ASL (using common sign vocabulary while minimizing fingerspelling). Participants understand they must share that the cancer screening appointment has been completed and its results with researchers for evaluation purposes, since the evaluation of the experimental arm’s intervention ended at the point of completed screening.

All participants receive tokens of appreciation. Two equal-sized financial gifts were originally approved by the IRB. However, as long-term retention in the study proved to be a concern, an alternate gifting strategy was requested and approved by the IRB: a smaller gift after baseline data collection ($25) and a larger one after completing the final assessment ($75) to recognize the greater value of their persistence in the study.

### Study Design

This study uses a parallel group, block-randomized design with 1:1 allocation stratifying 200 participants who are DDBHH by age and sex, with 100 participants assigned to the ASL-CHN intervention and 100 to standard of care.

All participants were confirmed as being nonadherent to at least 1 of 5 age-appropriate cancer screening guidelines recommended by the United States Preventive Services Task Force (USPSTF [11]). Nonadherence to the guidelines was confirmed during the recruitment process and again during the gathering of baseline data postrandomization. If a randomized participant either realized that they had indeed already been screened for all recommendations or were screened during the interval between recruitment and baseline data collection, they were removed from the study. In these instances of removal, those participants were moved to a separate database for future studies that they may be eligible for.

No significant protocol modifications have been implemented since trial commencement.

### Sample Recruitment

Multiple recruitment strategies were used to attract a diverse sample of individuals who are DDBHH nationwide. One strategy was to contact individuals who are DDBHH who had participated in prior studies conducted by this paper’s senior Gallaudet University researcher. The prior study participants were reminded of their past study participation and asked if they would be willing to learn about a new research study that also had the potential to benefit the DDBHH community.

Another recruitment strategy was to work with community leaders across the nation to set up local recruitment events. A third was to attend major community events that attracted large groups from the community where study recruitment could be efficiently conducted. The recruitment and consenting process used for all of these strategies were comparable.

Those who indicated their interest in participating were asked a set of questions to determine their eligibility for the new study. Once their eligibility was confirmed, they were told a follow-up contact would be made to formally enroll them in the study. During the follow-up enrollment conversation, the participants’ eligibility was reconfirmed and then they were enrolled in the study and randomly assigned to a study arm based on their sex and age as previously decided by the randomization scheme.

### Study Arms

#### Intervention Arm

In the intervention arm with videoconferencing, specially trained ASL-CHNs meet with participants over a period of up to 11 months. This extended timeframe is deliberately designed to accommodate the often-lengthy scheduling process for certain cancer screenings, which can take several months from initial referral to completion due to limited availability of specialists, preparation requirements, and health care system delays.

The intervention begins with an initial meeting to complete the consenting and randomization processes, gather baseline data, work with the participant to select the screening they would like to accomplish first, and provide directions on how to proceed with that screening. During the second meeting, about 2 weeks later, the ASL-CHN determines whether the screening has occurred or is scheduled and identifies any barriers encountered in making appointments or accomplishing the screening. If problems were encountered, the ASL-CHN engages in role-playing related to both the specific problem and commonly encountered problems during health care interactions.

All participants receive at least 4 months of intervention support. For those who have not completed screening by this 4-month assessment point, the study provides up to 7 additional months of support (for a total of up to 11 months). Throughout this period, the ASL-CHN continues to meet with participants to address emerging barriers, provide coaching through role-playing exercises, and offer support until screening is accomplished or until the maximum 11-month timeframe is reached.

#### Standard of Care Arm

The standard of care arm receives basic information about age-appropriate cancer screenings that have not been completed according to the recommended guidelines. The staff meet with standard of care participants through video calls. They help the participants select which screening the participant would like to accomplish, but without the navigation support offered to the experimental arm participants. They are asked to report back when they accomplish their screening.

If after 4 months in the standard of care arm they still have not scheduled their screening, they are recorded as “failed to be screened.” Participants remain blinded to their original group assignment, with measures in place to prevent cross-contamination despite the small DDBHH community. They are then told that they can be admitted to the other study arm (without any specific details about the difference between the 2 study arms), if they would like to again try to accomplish their screening.

**Figure 1:**
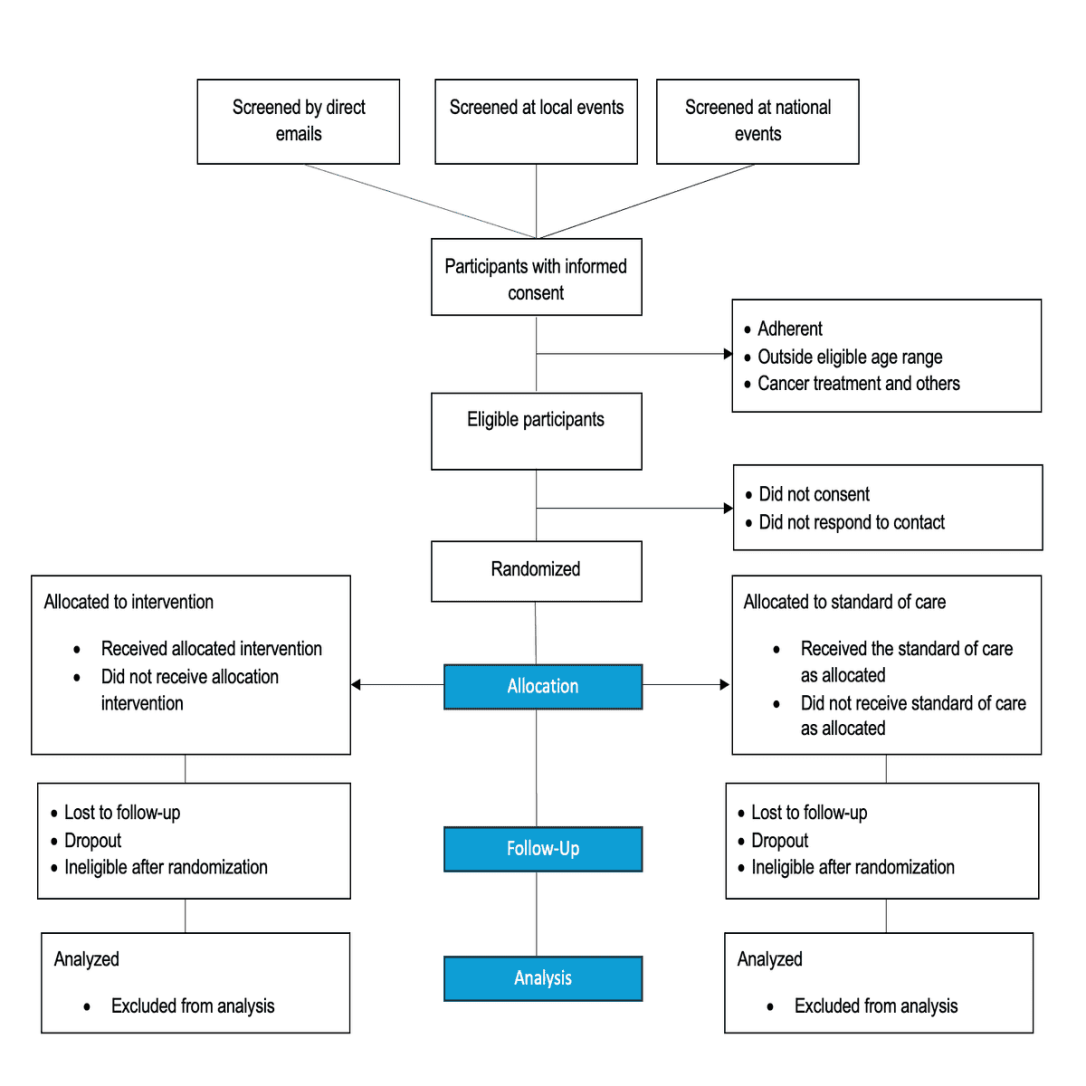
CONSORT (Consolidated Standards of Reporting Trials) diagram of the randomization process.

A CONSORT (Consolidated Standards of Reporting Trials) diagram of trial enrollment is provided in Figure 1.

### Study Integrity

The protocol implements several mechanisms to ensure study integrity and participant engagement. CHNs conduct comprehensive needs assessments with intervention participants, creating customized documentation of health care goals and questions. Both groups receive reminder messages before scheduled study visits, though only intervention participants receive screening appointment reminders. Follow-up data collection occurs at 2 weeks, 2 months, and 4 months, with standardized measures of cancer knowledge and patient-physician communication compared to baseline.

The study designates participants as “successful” if they complete recommended screenings during the trial period, with documentation required as evidence. To manage potential community interactions among this low-incidence population, CHNs and participants are instructed not to discuss their participation outside the study team, with provisions for reassignment if working relationships prove unsuccessful or conflicted.

To streamline the study’s focus, participants with multiple screening recommendations initially choose just 1 screening guideline, though they are encouraged to pursue additional screenings if the first is completed. Upon conclusion, a clinical research coordinator conducts videophone exit interviews and debriefing sessions with all participants to gather final insights about their experience.

### Setting and Participants

The study is conducted at Gallaudet University, with participants recruited across the nation. We will enroll a minimum of 200 study participants who have been defined as not having had 1 or more recommended screenings during the past year in the clinical trial. Each participant who is DDBHH must meet all of the following eligibility criteria: (1) be age-eligible for cancer screening, (2) have been born deaf or experienced loss of hearing by age 13 years (defined as “early deafness”), (3) be unaligned with their age- and risk-appropriate cancer screening during the past year, (4) have no contraindications to cancer screening, and (5) be available to participate during the study period. We will use USPSTF guidelines for breast, lung, prostate, colon, and cervical cancers to evaluate for possible contraindications and determine eligibility [11].

### Outcome Measures

Bilingual ASL-English measures (eg, sociodemographics, cancer screening knowledge, and patient-physician communication) will be administered at screening and again after the fourth month or by 11 months for comparison.

The primary outcome is completion of age- and risk-appropriate recommended cancer screening, which is a binary variable measured at the closeout follow-up for breast, cervical, colon, and lung cancer. For prostate cancer screening, the primary endpoint is participation in shared decision-making with the clinician.

The secondary outcome is patient-physician communication measured by the National Cancer Institute’s Health Information National Trends Survey (NCI-HINTS) Patient Centered Communication questionnaire that was validated in ASL [12]. The set of items for the Patient Centered Communication score are outlined in Textbox 1.

**Items for the Patient Centered Communication score.**

How often did the doctors, nurses, or other health care professionals you saw during the past 12 months do each of the following:

a) Give you the chance to ask all the health-related questions you had?

b) Give the attention you needed to your feelings and emotions?

c) Explain things in a way you could understand?

d) Spend enough time with you?

c) Involve you in decisions about your health care as much as you wanted?

d) Make sure you understood the things you needed to do to take care of your health?

e) Help you deal with feelings of uncertainty about your health or health care?

The tertiary exploratory outcome is cancer knowledge and includes two questions:

1. It seems like everything causes cancer.
2. How much do you think that gaining weight in adult life can influence whether or not a person will develop cancer?

### Analysis Plan

All analyses will be conducted using SAS 9.4, with a 2-sided *P* value <.05 considered statistically significant. The DDBHH sample size sufficiently powers the detection of differences in the primary study outcomes. Based on a 2-sided Z-test with unpooled variance and a significance level (α) of .05, our study sample of 200 participants with 100 in each group will achieve at least 80% power to detect a difference in proportions of 14%-17% in the group proportions of adherence (ie, almost 82%-92% adherence in the intervention group, assuming 65%-78% of the eligible participants in the standard of care group have obtained age- and risk-appropriate cancer screening at follow-up) based on previous studies. Univariate statistics for baseline characteristics will be computed with stratification by group, as will patient-physician communication and cancer knowledge at baseline and follow-up. Bivariate analyses will examine group differences in patient characteristics. We will primarily use an “intent-to-treat” approach, using multivariable logistic regression to evaluate whether the recommended cancer screening rates are higher in the intervention group than in the standard of care group. Though we do not expect strong clustering effects with CHNs, we will account for potential clustering using generalized estimating equations, with regression models including group assignment and adjusting for relevant covariates.

If a sufficient number of participants cross over from the standard of care arm to the intervention group, we will conduct sensitivity analyses to assess whether they performed better than the standard of care group. Despite efforts to maximize retention, we anticipate approximately 25% attrition, which may introduce bias. To address this, we will determine if dropouts differ between randomized groups, adjust for any resulting imbalances in baseline covariate distributions, and perform sensitivity analyses under different assumptions (missing completely at random, participants who dropped out would have been responders, or participants who dropped out would have been nonresponders).

If more than 10% are missing endpoint or covariate data, we will consider multiple imputation under the missing at random assumption and conduct sensitivity analysis with the imputed data. This comprehensive approach will help ensure robust evaluation of intervention effects while accounting for potential sources of bias in our study design. Our analytical strategy balances methodological rigor with practical considerations related to participant retention and data completeness, allowing for reliable conclusions about the effectiveness of our intervention for improving cancer screening rates.

## Results

Participant recruitment commenced in July 2023 and continued until August 2025. Follow-up data collection has commenced and will be completed by February 2026, with results expected in March 2026.

While the clinical trial study is ongoing, more than 75% of the target enrollment has been achieved. Preliminary data indicate that the intervention group is consistently outperforming the standard of care group in cancer screening adherence, supporting the study hypothesis that ASL-CHNs are effective in promoting cancer screening adherence among previously nonadherent participants who are DDBHH.

## Discussion

### Anticipated Findings

The need to promote cancer screening adherence among adults who are DDBHH is significant. Our ASL-CHN intervention responds to the findings that individuals who are DDBHH face complex systemic, attitudinal, communication, and personal-level barriers when seeking cancer screening [1]. By using CHNs who are ASL-proficient and share lived experiences with the DDBHH community, the intervention addresses specific challenges identified in our needs assessment, including ineffective patient-physician communication, limited access to cancer information in ASL, misconceptions about medical procedures, difficulties navigating insurance systems, and intersectional barriers for individuals with multiple marginalized identities. The intervention focuses on training CHNs to provide appropriate support, clear explanations of cancer screening terminology using ASL, assistance with health care system navigation for cancer screenings, and emotional support—ultimately working to reduce cancer screening gaps documented among individuals who are DDBHH.

By engaging patients who are DDBHH via ASL-fluent navigators, the ASL-CHN intervention offers a scalable, participant-centered, and highly accessible solution. The language concordant communication included in ASL-CHN programming is intentionally designed to help participants focus on getting screened for cancer without feeling marginalized by the health care system. This careful approach is intended to mitigate health care mistrust, which is a major impediment to long-term engagement with preventive care in this population [13,14].

Preliminary evidence suggests that the ASL-CHN intervention is promising in promoting cancer screening and adherence among adults who are DDBHH [1]. The integration of personalized navigation with ASL-CHNs addresses key limitations of previous cancer screening interventions, which often lacked the accessible support necessary to sustain long-term health care engagement. Our approach in this randomized controlled trial aligns with recent studies that have highlighted the importance of combining Zoom and videophone technology with accessible human interaction to promote engagement and adherence in preventive health programs.

Understanding the mechanisms of action underlying the efficacy of ASL-CHN is highly significant. In this study, we are examining how our intervention improves cancer screening adherence through enhanced patient-centered care and strengthened shared decision-making between patients who are DDBHH and their physicians. By addressing communication barriers and empowering patients with accessible health information, ASL-CHN creates opportunities for meaningful dialogue about screening options, personal preferences, and concerns. These data will inform further refinement and optimization of the ASL-CHN program to maximize patient engagement in cancer prevention decisions.

This study has a few limitations worth acknowledging. We face retention challenges due to participants’ limited understanding of clinical trial processes and procedures. Additionally, our requirement for ASL fluency restricts our ability to include the broader DDBHH population who may use different communication methods. Our focus on only 5 major cancer screening types (breast, cervical, colorectal, lung, and prostate) may not address other important cancer prevention needs.

However, if successful in promoting cancer screening adherence for the 5 major cancer screening types, the ASL-CHN intervention could provide a practical, scalable option that health care providers can recommend to patients who are DDBHH who are not screening-adherent and may benefit from navigator support. Because early detection has been shown to reduce cancer mortality, the ASL-CHN intervention has the potential to play a critical role in improving health outcomes in this underserved population.

### Conclusions

The availability of an effective, accessible ASL-CHN service has the potential to shift future research that improves health outcomes, which helps lower the cost of associated health care and promote longer lives among people who are DDBHH who use ASL. We envision an ASL-CHN functioning as a patient navigator who is able to promote cancer screening adherence among people who are DDBHH. Patients who are DDBHH may experience even greater benefit from CHNs who are fluent in ASL, since so few clinicians are available and competent when caring for patients who are DDBHH. This should help support improvements in health care with regards to availability, affordability, adequacy, and administrative simplicity. It should also increase engagement with and adherence to cancer screenings, thereby improving health outcomes for people who are DDBHH who use ASL. To achieve this vision, cancer screening guidelines as well as navigation tools accessible in ASL are needed as resources for ASL-CHNs who encounter patients who are DDBHH in their care.

## Appendix

Multimedia Appendix 1 Peer review report by ZRG1 MOSS-*t* (54) Center for Scientific Review Special Emphasis Panel UNITE Transformative Research to Address Health Disparities and Advance Health Equity at Minority Serving Institutions (U01) (National Institutes of Health, USA).

## Abbreviations

ASL: American Sign Language
ASL-CHN: ASL-fluent community health navigator
CHN: community health navigator
CHW: community health worker
CONSORT: Consolidated Standards of Reporting Trials
DDBHH: deaf, deafblind, and hard of hearing
IRB: institutional review board
USPSTF: United States Preventive Services Task Force
