# Supplementary material for: Using American Sign Language–Fluent Community Health Navigators to Advance Cancer Screening Adherence through Videoconferencing With Deaf, Deafblind, and Hard of Hearing Adults: Protocol for a Randomized Controlled Trial
Source: JMIR Res Protoc. 2025 Sep 9;14:e65078. doi: 10.2196/65078 (PMC12457851; doi:10.2196/65078)
Supplement: Multimedia Appendix 1 [file resprot_v14i1e65078_app1.pdf]

SUMMARY STATEMENT

PROGRAM CONTACT: **(Privileged Communication)** ***Release Date:*** **08/15/2021**

[redacted] ***Revised Date:***

Application Number: **1 U01 DE031620-01**

Principal Investigator

KUSHALNAGAR, POORNA

Applicant Organization: GALLAUDET UNIVERSITY

Review Group:	**ZRG1 MOSS-T (54)**<br>**Center for Scientific Review Special Emphasis Panel**<br>**UNITE Transformative Research to Address Health Disparities and Advance Health Equity at Minority Serving Institutions (U01)**
Meeting Date:	**07/29/2021**
Council:	**AUG 2021**
Requested Start:	**09/01/2021**
RFA/PA:	**RM21-022**
Dual IC(s):	**RM, OD**
Project Title:	**Using technology-enhanced approaches to advance cancer health equity among diverse deaf, deafblind, and hard of hearing populations.**
SRG Action:	[redacted]
Next Steps:	**Visit https://grants.nih.gov/grants/next_steps.htm**
**Human Subjects:**	**30-Human subjects involved - Certified, no SRG concerns**
**Animal Subjects:**	**10-No live vertebrate animals involved for competing appl.**
**Gender:**	**1A-Both genders, scientifically acceptable**
**Minority:**	**1A-Minorities and non-minorities, scientifically acceptable**
**Age:**	**3A-No children included, scientifically acceptable**

[redacted]

1U01DE031620-01 Kushalnagar, Poorna

RESUME AND SUMMARY OF DISCUSSION: The goal of this U01 application is to develop educational content and cancer screening resources for Community health workers (CHWs) working with the deaf, deafblind, and hard of hearing (DDBHH) population, who would function as cancer health navigator to promote cancer screening adherence and reduce health disparity among DDBHH people. During the discussion, reviewers noted that the study is built upon the investigative team's prior work where they have developed a technology platform, specifically SNAP (the System for Navigational Assistance for Patients), a technology to support cancer patient navigators, and REPEAT (Realizing Enhanced Practice through Adaptive Tutoring), a tool to build virtual guided practice environments for developing skills in clinical communication domains. The research team has complementary expertise in health promotion with DDBHH, lived experience, technology, and mobile app development, health management, health disparities, and research with the target population. The study population is highly underserved and successful completion of the study would have a transformative impact that will allow CHWs to educate the DDBHH population on cancer preventive services. The study is innovative and the team has already built and tested the technology and has an excellent dissemination plan. However, some weaknesses were identified especially in the approach. Reviewers noted that it is unclear how the clinicians will benefit from this intervention and how metrics will be assessed for clinical providers beyond the educational videos. The sustainability factor is unclear in terms of workflow and team dynamics with CHWs fluent in ASL working in-person vs. virtual. It is also unclear how the CHWs will be culturally responsive to different racial/ethnic and cultural groups. It is unclear what kind of culturally appropriate content will be developed. Some reviewers noted that there is no engagement of community stakeholders beyond the research team. However, others noted that the investigators bring in important life experience and have a vast amount of experience working with this community. It should be noted that while in the beginning some reviewers had more concerns, but following discussion, everyone agreed on the strengths and weaknesses, and most weaknesses were considered as missed opportunities and addressable. Thus, the overall enthusiasm of the review panel remained high for this highly significant application.

DESCRIPTION (provided by applicant): About 500,000 use American Sign Language (ASL). Among DDBHH people who use ASL, there are documented reports of difficulties in understanding cancer health information in print, accessing clinical services for screenings, and understanding and following treatment-related directives if the screening results warrant a cancer diagnosis. These difficulties contribute to the health inequity that we observed in the DDBHH populations. Community health workers have proven their value with hearing patients, so it is reasonable to expect that ASL- proficient community health workers (CHW) will have the same potential to identify and resolve DDBHH people's barriers to receiving optimal care for cancer prevention and screening. We envision an ASL-proficient CHW, functioning as a cancer health navigator, who is able to promote cancer screening adherence and reduce health disparity among DDBHH people. This research will build on the team's current NIH-funded research and recently developed technology platform, specifically SNAP (the System for Navigational Assistance for Patients), a technology to support cancer patient navigators, and REPEAT (Realizing Enhanced Practice through Adaptive Tutoring), a tool to build virtual guided practice environments for developing skills in clinical communication domains. Use of SNAP and REPEAT will provide an advanced starting point that allows us to focus on the specific training and community application needs of the DDBHH community, rather than new technology development. Our ability to address these specific needs and to appropriately deliver and evaluate the interventions is supported by a highly interdisciplinary team, spanning expertise in DDBHH health disparity, disability studies,

public health, health informatics, cultural anthropology, human factors engineering, and computer science

PUBLIC HEALTH RELEVANCE: Community health workers and navigators (CHWs) can play a role in public education and outreach for health equity within the deaf, deafblind, and hard of hearing (DDBHH) community. In addition, because many cancer screening guidelines vary based on family history and personal risk factors, CHWs are in a position to provide information about screening services that helps to improve DDBHH patients’ cancer knowledge and risk perception, and adherence to screening guidelines.

CRITIQUE 1

Significance: 2
Investigator(s): 2
Innovation: 3
Approach: 3
Environment: 3

Overall Impact: This is a highly significant area of focus with a highly underserved population with a strong team and environment. However, the approach is not innovative- community health workers and patient navigators are well-established as effective in improving cancer screening, follow up and adherence to treatment. ASL proficiency while super important does not make a navigator intervention model innovative. Patient navigation is a “band aid” approach to a broken system and there is a missed opportunity to add some implementation science measures to help contribute to the transformative nature of this proposal- as patient navigators and chw’s are approaches that are difficult to sustain without grant funding.

1. Significance:

Strengths

- Significant area of focus on cancer disparities amongst DDBHH persons.

Weaknesses

- None noted.

2. Investigator(s):

Strengths

- Outstanding team with strong expertise in DDBHH, health disparities, health informatics, human factors engineering and computer science.

Weaknesses

- None noted.

3. Innovation:

Strengths

- It is modestly innovative that this team is leveraging the technological platforms they have built to help DDBHH persons and to support ASL fluent CHWs in navigating DDBHH persons through cancer screening and follow up

Weaknesses

- Patient navigators are not transformative intervention models- but the population of focus helps improve the transformative nature
- Cme videos and practice encounters are important for training but not innovative

4. Approach:

Strengths

- The team has already built and tested the technology
- Using the technological platforms with navigators and virtual navigator access holds promise to help support potential sustainment of this intervention beyond the study period

Weaknesses

- The approach to use the technological platforms to assist CHW's fluent in ASL to navigate patients falls short of being able to tease out what could actually be learned from CHW's fluent in ASL to scale beyond individual chw/navigator encounters- regardless of if they are virtual or in person.
- It is not clear how chw's will virtually navigate the participants to resources that are non-local to the navigator/chw themselves- the chw's/navigators have to have intimate knowledge of the communities in which they are navigating patients and it is not clear how this will be done virtually. The navigation seems to be solely focused on the clinical care encounters and not on assisting with the multiple other barriers to care which include social determinants of health and experience of racism /discrimination etc.
- It is not clear how beyond the cme videos the clinicians will benefit from this intervention and how metrics will be assessed for clinical care providers.
- There is a missed opportunity to assess implementation factors in this study so that sustainability and factors such as workflow and team dynamics with CHWs fluent in ASL that are working in person vs. virtual can be addressed.
- This is a national virtual study- and it is not clear how the chw/pn's will be culturally responsive to the different racial/ethnic and cultural groups beyond being a person from the DDBHH community who is ASL proficient.

5. Environment:

Strengths

- Excellent environment.

Weaknesses

- While the members of the research team are from the DDBHH community, the level of community stakeholder involvement outside of this team is not well detailed.

Study Timeline

Strengths

- Adequate timeline

Weaknesses

- None noted.

Protections for Human Subjects:

Unacceptable Risks and/or Inadequate Protections

- The details for protections are underdeveloped. This is a rct and a clinical trial- there should be a dsmp with more detail

Data and Safety Monitoring Plan (Applicable for Clinical Trials Only):

Unacceptable

- There should be a more detailed dsmp - this is a rct with virtual and in person navigation components.

Inclusion Plans:

- Sex/Gender: Distribution justified scientifically
- Race/Ethnicity: Distribution justified scientifically
- For NIH-Defined Phase III trials, Plans for valid design and analysis: N/A
- Inclusion/Exclusion Based on Age: Distribution justified scientifically

Vertebrate Animals:

Not Applicable (No Vertebrate Animals)

Biohazards:

Not Applicable (No Biohazards)

Resource Sharing Plans:

Acceptable

Authentication of Key Biological and/or Chemical Resources:

Not Applicable (No Relevant Resources)

Budget and Period of Support:

Recommend as Requested

CRITIQUE 2

Significance: 2
Investigator(s): 2
Innovation: 1
Approach: 3
Environment: 1

Overall Impact: Among DDBHH people who use ASL, there are documented reports of difficulties in understanding cancer health information in print, accessing clinical services for screenings, and understanding and following treatment-related directives if the screening results warrant a cancer diagnosis.

Community health workers have proven their value with hearing patients, so it is reasonable to expect that ASL- proficient community health workers (CHW) will have the same potential to identify and resolve DDBHH people's barriers to receiving optimal care for cancer prevention and screening. The applicants propose an ASL-proficient CHW, functioning as a cancer health navigator, who is able to promote cancer screening adherence and reduce health disparity among DDBHH people.

This research will build on the team's current NIH-funded research and recently developed technology platform, specifically SNAP (the System for Navigational Assistance for Patients), a technology to support cancer patient navigators, and REPEAT (Realizing Enhanced Practice through Adaptive Tutoring), a tool to build virtual guided practice environments for developing skills in clinical communication domains.

Significance:

About 500,000 use American Sign Language (ASL). Among DDBHH people who use ASL, there are documented reports of difficulties in understanding cancer health information in print, accessing clinical services for screenings, and understanding and following treatment-related directives if the screening results warrant a cancer diagnosis.

Less than 30% of the Deaf men sample reported feeling always or often engaged in decision making with their doctors. In another recent study, deaf mid-to-older adults with smoking history were significantly less likely to ask about lung cancer test if they did not use an interpreter at medical visits. Deaf young adults were significantly less likely to have heard of HPV (Human papillomavirus infection) and less likely to perceive that HPV vaccine is effective in preventing cervical cancer compared to hearing peers. In this deaf young adult sample, only 22.1% said their doctors recommended them to get a HPV vaccine. In another study from PI Kushalnagar's lab, deaf women continue to be at disparity for cervical and breast cancer screening compared to hearing women.

Strengths

- Strong preliminary data.
- Transformative in that it allows CHW to educate a population that did not fully leverage cancer preventative services.

Weaknesses

- None noted.

2. Investigator(s):

Strengths

- Poorna Kushalnagar: As a deaf investigator fluent in American Sign Language and English, she dedicated to the advancement of scientific knowledge related to health outcomes in deaf, deafblind, and hard of hearing (DDBHH) people. She has considerable experience conducting mixed methods, qualitative, human-computer interaction, and quantitative research with DDBHH people.
- She has served as the principal investigator of past and current federally-funded grants, 1) an NIH-R15 grant project on the national trends of DDBHH signers' use of the Internet for cancer health-related purposes, 2) an NIH-R01 grant project on DDBHH -related health outcomes, 3) two diversity grants to support DDBHH predoctoral students, and 4) some administrative grants to support DDBHH sexual/gender minority research, PROMIS Deaf Profile- Cognitive Concerns research with DDBHH older adult, and DDBHH caregivers who care for loved ones with ADRD.
- She provided support and leadership to recruit over 2,500 hard-to-reach participants and produced over 40 manuscripts in five years.
- Christopher J. Moreland: As a board-certified deaf physician in academic internal medicine, he has long explored health disparities affecting underserved populations using a language other than English, including the deaf and hard of hearing community. He will serve as a clinician co-investigator on the U01 project if funded.
- He has also led and collaborated on research with colleagues from Gallaudet University and the Association of American Medical Colleges to describe the population of physicians and healthcare professionals with disabilities. He will be appointed Clinical Co-Director, Center for Deaf Health Equity Gallaudet University in August 2021.
- Rupa S. Valdez: Dr. Valdez's research integrates qualitative and quantitative methods from multiple disciplines to characterize patients' health management practices across patients, informal caregivers, clinicians, and healthcare professionals across home, community, and clinical environments. Based upon these assessments, my work seeks to design and evaluate clinical and consumer health information technology interventions.
- Drs. Valdez and Kushalnagar have a history of collaboration at the intersection of health IT and health equity for the disability community, with a recently published manuscript in the Journal of the Medical Informatics Association focused on this topic.
- Strong research team with a history of previous collaboration
- Diverse investigator team that includes PhD researchers, a clinical research physician and computer scientist.

Weaknesses

- None noted.

Innovation:

Strengths

- Community health workers have proven their value with hearing patients, so it is reasonable to expect that ASL-proficient community health workers (CHW) will have the same potential to identify and resolve DDBHH people's barriers to receiving optimal care for cancer prevention and screening. We envision an ASL-proficient CHW, functioning as a cancer health navigator, who is able to promote cancer screening adherence and reduce health disparity among DDBHH people. Indeed, DDBHH people may experience even greater benefit from culturally and

linguistically aligned CHWs, since so few clinicians are available and competent when caring for DDBHH patients. CHWs who are DDBHH and fluent in ASL could help assure that ASL interpreters are delivering the clinician's information clearly to the DDBHH patient.

- The web- and mobile-based *CHW-ASL* tool has two parts: 1) fully accessible learning curriculum for CHWs and 2) virtual guided practice environments to support CHWs' patient-centered navigation work and clinicians' communication with DDBHH patients. Use of these components in *CHW-ASL* tool will provide an advanced starting point that allows us to focus on the specific training and community application needs of the DDBHH community, rather than new technology development that will require higher costs. Our ability to address these specific needs and to appropriately deliver an innovative, accessible technology that has potentials to significantly impact and transform health equity among medically underserved nonadherent individuals who are DBDHH and use ASL. This impactful work is supported by a highly interdisciplinary team, spanning expertise in DDBHH health disparity, medicine, public health, health informatics, human factors engineering, and computer science.
- This solution promotes CHWs' health literacy regarding the problems and barriers that DDBHH people face in engaging with, understanding, and adhering to cancer screening and prevention. The solution also provides insights into CHWs' knowledge on how to navigate DDBHH people through the healthcare system for cancer screening and adherence. This work has never been done before, but literature shows promise that the combination of the *CHW-ASL* technology and ASL-fluent CHWs is the best, low-cost, and rapid approach to reducing the longstanding disparity in cancer health among DBDHH individuals in the U.S.
- Developing a learning content and cancer screening resource for CHWs who are ASL-fluent and culturally competent to work with DDBHH population.
- Creating a web- and mobile-based CHW tool to support CHWs in providing DDBHH patient navigation service.
- Implementing a transformative CHW intervention that is fully accessible and culturally relevant to DDBHH people's experiences with cancer screenings and prevention.

Weaknesses

- None noted.

4. Approach:

Aim 1: Co-create a learning content for a virtual community health worker service program for DDBHH CHWs and complementary training program for clinicians who work with DDBHH patients.

Aim 2: Assess the efficacy of a web- and mobile-app based tool in CHW's perceived confidence in reducing health disparity for cancer screening.

Aim 3: To implement and evaluate virtual CHW service's successful promotion of cancer screening adherence by DDBHH people who use ASL.

Sample size calculation: A statistical power analysis using G*Power 3.1.9.7 was performed for sample size estimation. A medium effect size (ES) was expected using Cohen's 1988 criteria of .5. With the other parameters of alpha = .05 and power = .90 the projected sample size of approximately 73 was produced. Thus, even with expected attrition, the proposed sample of 100 in each group is more than suitable for the study objectives

An "intent-to-treat" (ITT) approach will be used.

Expected outcomes

- At least 30% increase over the baseline for cancer screening adherence;
- At least 30% increase over the baseline for cancer knowledge; and
- At least 30% increase over the baseline for having a regular healthcare provider or improvement in patient-physician communication.

Dissemination

- Scientific Presentations
- Community-Based Presentations.
- Publications

Strengths

- Strong experimental approach.
- Strong data safety monitoring.
- Excellent dissemination plan.

Weaknesses

- None noted.

5. Environment:

Strengths

- Gallaudet University is one of the premier institutions for deaf and hard of hearing students.
- The research arrangements between Gallaudet, UVA, Sam Houston State and Starship are good.

Weaknesses

- None noted.

Study Timeline

Strengths

- Clearly delineated with obtainable milestones.

Weaknesses

- None noted.

Protections for Human Subjects:

Acceptable Risks and/or Adequate Protections

- Appropriate

Data and Safety Monitoring Plan (Applicable for Clinical Trials Only):

Acceptable

Inclusion Plans:

- Sex/Gender: Distribution justified scientifically

- Race/Ethnicity: Distribution justified scientifically
- For NIH-Defined Phase III trials, Plans for valid design and analysis: Not applicable
- Inclusion/Exclusion Based on Age: Distribution justified scientifically

Vertebrate Animals:
Not Applicable (No Vertebrate Animals)

Biohazards:
Not Applicable (No Biohazards)

Resource Sharing Plans:
Not Applicable (No Relevant Resources)

Authentication of Key Biological and/or Chemical Resources:
Not Applicable (No Relevant Resources)

Budget and Period of Support:
Recommend as Requested

CRITIQUE 3

Significance: 2
Investigator(s): 2
Innovation: 2
Approach: 4
Environment: 1

Overall Impact: This proposal is to develop a learning content and cancer screening resource for Community health workers (CHWs) working with the deaf, deafblind, and hard of hearing (DDBHH) population, as well as test the feasibility and acceptability of the web- and mobile-based CHW-ASL tool. The premise for the study is that CHWs can play an important role in promoting public health and health equity in the DDBHH community and that emerging technology intervention can be feasible and effective in reducing health disparities in this underserved population. The investigative team and environment are great, with strong preliminary work in this area of inquiry, and addresses an important healthcare issue in an underserved population. The research team has complementary expertise in health promotion with DDBHH, patient education, technology and mobile app development, health management, health disparities and research with the target population. The innovation for this study lies in the potential of developing a cutting-edge web- and mobile-based CHW-ASL tool in three phases. Of major concern was the under-developed research plan for aim 3. Other weaknesses are either moderate or minor concerns in nature. Overall, this proposal has merits and innovative ideas.

Although there are some concerns in the Significance and Approach section, this study has great potential for success and impact. The transformative potential of this application is promising.

Significance:

Strengths

- Developing and testing novel and scalable technology-based interventions to in DDBHH population could reduce cancer-related morbidity and costs, as well as address health disparities and equity among this underserved population.
- The pilot intervention for phase 3 with emerging technology is on the cutting edge and has potential to disseminate to a larger scale. Thus, the transformative potential of this application is promising.
- The newly developed learning content and resources, as well as the technology-based intervention approach, may be cost-effective in the healthcare settings and if they are proven to be feasible and effective, it would be transformative and clinically meaningful for DDBHH population.

Weaknesses

- The literature reviewed is limited to the PI's own work among black DDBHH. Unclear what has been done with DDBHH from other race/ethnicity (moderate).
- There are many types of cancers and different approaches for cancer screening. Unclear if the research team wants to include all cancer types and how to deal with the heterogeneity among them (moderate).
- Details of the role of CHW in helping DDBHH people is warranted (minor).
- Sex differences during the content development and the intervention efficacy will not be examined in this study (moderate).
- Unclear what type of culturally appropriate solutions or contents will be developed (moderate).

2. Investigator(s):

Strengths

- The PI is an established investigator and has an excellent record of NIH funding and publications in the field of DDBHH, health disparities, patient education and patient-physician communication.
- The team has complementary expertise in deaf physician, technology and mobile app development, health management, health disparities, and clinic trials.
- All Co-Is can be good additions to the research team.

Weaknesses

- Lack of expertise in biostatistics.

3. Innovation:

Strengths

- Promoting health in DDBHH people to resolve health disparities through emerging technology is rare.

- The study fills a gap in developing a learning content and cancer screening resource for CHWs working with DDBHH population.
- The technology-based intervention approach in DDBHH people itself has been less studied, so the development of such intervention program, namely web- and mobile-based CHW-ASL tool, and test its feasibility is novel.

Weaknesses

- None noted.

4. Approach:

Strengths

- The research team has built up its experience with developing such a technology platform.
- The procedures for aim 1 and aim 2 are detailed.
- Sample size for aim 3 seems sufficient.
- The research team has demonstrated its capability in recruiting sufficient participant in predetermined timelines.

Weaknesses

- Rationale of using focus groups as opposed to individual in-depth interview in phase 1 is needed (minor).
- Qualitative data analysis (e.g., data triangulation) in different phases (e.g., focus groups, semi-structured intervention data sorting, and analysis) is warranted (major).
- One diagram or figure may help better illustrate the process of developing the learning content and web- and mobile-based CHW-ASL tool (minor).
- The rationale for the intervention length of phase 3 is omitted. Better justification could be provided for this. Unclear of the retention rate in phase 3, although the team indicates to have extensive experience with DDBHH community and predict < 25% attrition rate. Also, intervention fidelity will be continuously monitored (moderate).
- Inclusion criteria are listed for DDBHH participants but not CHWs. Also, exclusion criteria should be included (minor).
- Unclear about the number of follow-ups in phase 3 (minor).
- Unclear how the research team will do to avoid data contamination across groups in phase 3 (minor).
- The analysis plans for aim 3 are not clear. For example, to examine the group differences of the outcomes in this study, mix models will be more appropriate than the linear regression model (moderate).
- No anticipated problems and strategies to resolve the issues/problems for the project? (moderate).
- Some typos (e.g., framework, extra spaces between words) can be found in this section (minor).

5. Environment:

Strengths

- The environment is generally strong and supportive.
- The research team will be able to recruit and train students from DDBHH backgrounds in biomedical and behavioral research.

Weaknesses

- Not sure how the researchers from Gallaudet University would collaborate with the Co-Is from several other institutions from other states (e.g., remote virtual meetings, teleconference, etc.) in this study, as the Overall Structure of the Study Team is not detailed with simple descriptions (minor).

Study Timeline:

Strengths

- The timeline is detailed.

Weaknesses

- A Gantt chart may work better in this case (minor).

Protections for Human Subjects:

Acceptable Risks and/or Adequate Protections

Data and Safety Monitoring Plan (Applicable for Clinical Trials Only):

Acceptable

Inclusion Plans:

- Sex/Gender: Distribution not justified scientifically
- Race/Ethnicity: Distribution justified scientifically
- For NIH-Defined Phase III trials, Plans for valid design and analysis: N/A
- Inclusion/Exclusion Based on Age: Distribution justified scientifically

Vertebrate Animals:

Not Applicable (No Vertebrate Animals)

Biohazards:

Not Applicable (No Biohazards)

Resource Sharing Plans:

Acceptable

Authentication of Key Biological and/or Chemical Resources:

Not Applicable (No Relevant Resources)

Budget and Period of Support:
Recommend as Requested

THE FOLLOWING SECTIONS WERE PREPARED BY THE SCIENTIFIC REVIEW OFFICER TO SUMMARIZE THE OUTCOME OF DISCUSSIONS OF THE REVIEW COMMITTEE, OR REVIEWERS' WRITTEN CRITIQUES, ON THE FOLLOWING ISSUES:

PROTECTION OF HUMAN SUBJECTS: ACCEPTABLE

INCLUSION OF WOMEN PLAN: ACCEPTABLE

INCLUSION OF MINORITIES PLAN: ACCEPTABLE

INCLUSION ACROSS THE LIFESPAN: ACCEPTABLE

COMMITTEE BUDGET RECOMMENDATIONS: The budget was recommended as requested.

Footnotes for 1 U01 DE031620-01; PI Name: Kushalnagar, Poorna

NIH has modified its policy regarding the receipt of resubmissions (amended applications).See Guide Notice NOT-OD-18-197 at https://grants.nih.gov/grants/guide/notice-files/NOT-OD-18-197.html. The impact/priority score is calculated after discussion of an application by averaging the overall scores (1-9) given by all voting reviewers on the committee and multiplying by 10. The criterion scores are submitted prior to the meeting by the individual reviewers assigned to an application, and are not discussed specifically at the review meeting or calculated into the overall impact score. Some applications also receive a percentile ranking. For details on the review process, see http://grants.nih.gov/grants/peer_review_process.htm#scoring.
